# Development of a novel real-time polymerase chain reaction assay for the quantitative detection of Nipah virus replicative viral RNA

**DOI:** 10.1371/journal.pone.0199534

**Published:** 2018-06-19

**Authors:** Kenneth S. Jensen, Ricky Adams, Richard S. Bennett, John Bernbaum, Peter B. Jahrling, Michael R. Holbrook

**Affiliations:** NIAID Integrated Research Facility, Ft. Detrick, Frederick, MD, United States of America; University of Texas Medical Branch at Galveston, UNITED STATES

## Abstract

Nipah virus (NiV) is a highly pathogenic zoonotic paramyxovirus that can result in severe pulmonary disease and fatal encephalitis in humans and is responsible for outbreaks in Bangladesh, Malaysia, Singapore, India and possibly the Philippines. NiV has a negative-sense RNA genome that contains six genes and serves as a template for production of viral mRNA transcripts. NiV mRNA transcripts are subsequently translated into viral proteins. Traditionally, NiV quantitative real-time reverse transcriptase polymerase chain reaction (qRT-PCR) assays have relied on using primer sets that amplify a target (N that encodes the nucleocapsid) within the coding region of the viral gene that also amplifies viral mRNA. Here we describe a novel one-step qRT-PCR assay targeting the intergenic region separating the viral F and G proteins, thereby eliminating amplification of the viral mRNA. This assay is more accurate than the traditional qRT-PCR in quantifying concentrations of viral genomic RNA.

## Introduction

Nipah virus (NiV) is a zoonotic virus that naturally infects Pteropid fruit bats and has been associated with outbreaks in humans in Malaysia, Bangladesh, India, and the Philippines [1–8]. NiV infection of humans can result in a severe pulmonary infection and neurological disease, and approximately 20% of survivors developing long-term neurological sequelae [9]. Patients infected with NiV can also experience either a late-onset or have a relapse of encephalitis months after the initial virus infection [10–12]. Recent NiV outbreaks have resulted in a case fatality rate ranging from 33% to 100% [3, 6, 13], with a cumulative case fatality rate around 56% [14].

**Fig 1:**
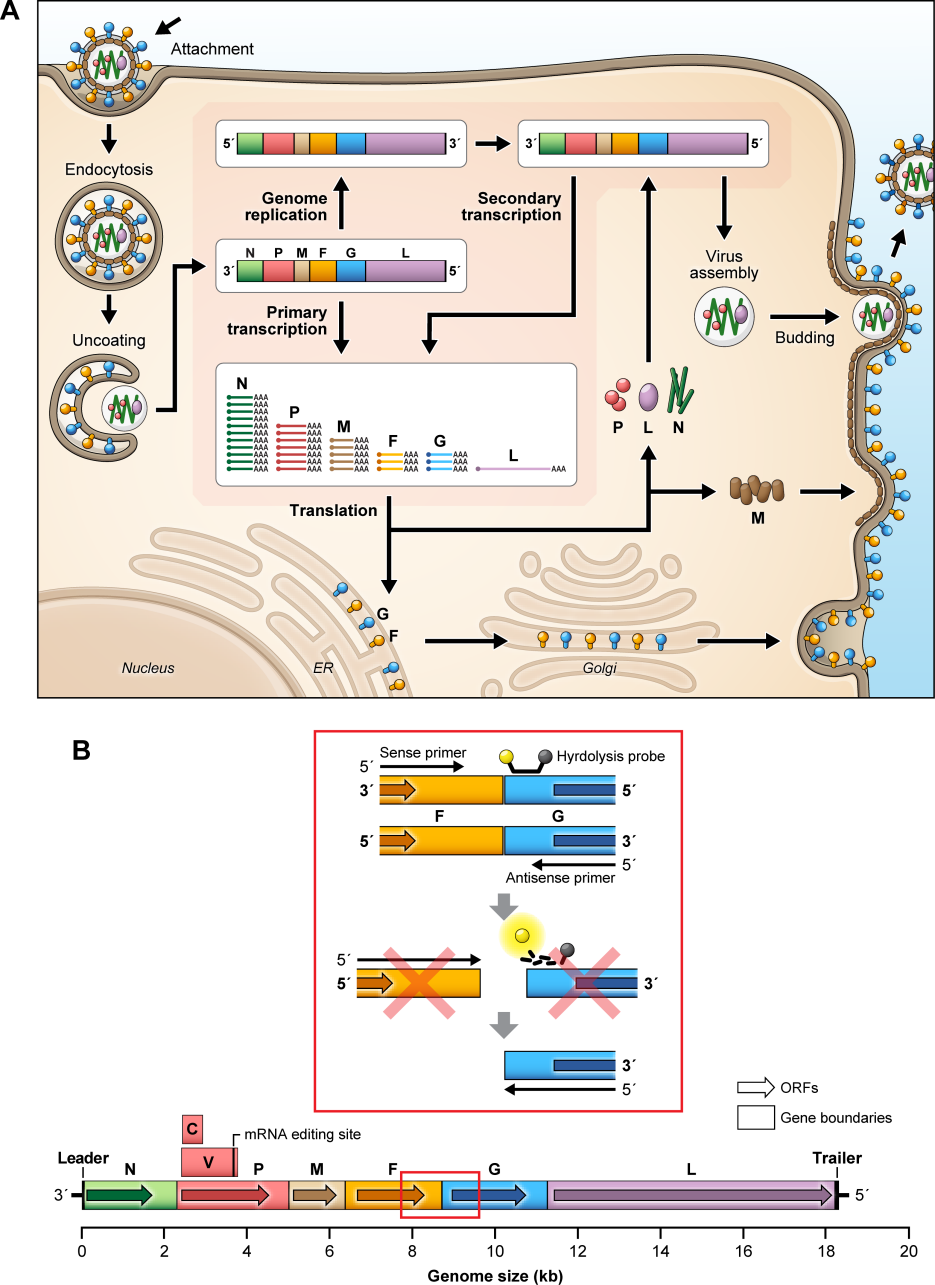
Nipah virus (NiV) replication and genome organization. (A) Nipah virus replication cycle. NiV enters a cell, where the viral genome is released, leading to the initiation of transcription and the accumulation of viral mRNA transcripts. In addition, the viral genome is transcribed into a full-length anti-genome, which is used to generate additional copies of the NiV genome. Viral mRNA transcripts are translated into viral proteins, leading to virion assembly, encapsidation, and virus release. (B) Schematic of NiV qRT-PCR assay. The NiV qRT-PCR assay was designed across the NiV F-G intergenic region. The forward primer binds to the untranslated region of the F gene. The reverse primer and hydrolysis probe bind to the 3’ region of the G gene, thereby preventing viral mRNA transcripts from providing false signals and artificially increasing viral RNA concentrations.

NiV is a negative-stranded, non-segmented RNA virus belonging to the genus *Henipavirus* in the family *Paramyxoviridae*. The genome of NiV is 18,246 nucleotides (nt), consisting of six genes encoding the nucleocapsid (N), phosphoprotein (P), matrix protein (M), fusion protein (F), glycoprotein (G), and the polymerase (L) that are separated by non-coding intergenic regions. Members of the genus *Henipavirus* have a genome that is distinct from most other paramyxoviruses, in that five of the six viral genes, not including the *L* gene, contain long untranslated regions (UTR) at the 3’ end of the viral mRNA transcripts (Fig 1A) [1, 2].

Upon virus infection of a cell, the viral genome serves as a template for transcription of viral mRNA, with transcription regulated by transcriptional start and stop sites at the 3’ and 5’ ends of each gene. During transcription, the polymerase complex (P and L proteins) polyadenylates each viral mRNA transcript, pauses at the gene stop signal, crosses the intergenic region, and reinitiates transcription at the next gene start signal [6]. During this process, some of the polymerase complexes will fall off the viral genome, resulting in a gradient of viral mRNA transcripts within a cell (Fig 1A). Published one-step assays to detect NiV are designed to target the viral N gene that is highly expressed during NiV replication [6, 15–17]. Targeting highly expressed genes can lead to an increase in assay sensitivity. However, the assay will not differentiate between the viral genome and viral mRNA transcripts in samples collected *in vitro* or *in vivo*, preventing accurate viral genome quantification.

Here we describe two novel assays designed to quantify the genome copy numbers of either NiV-Malaysia (NiV-M) or NiV-Bangladesh (NiV-B) isolates in a sample, independent of viral mRNA transcript concentrations. Genome copies from each sample were compared to measured plaque forming units (PFU) and viral particles to determine genome:PFU:particle ratios. The one-step quantitative reverse-transcriptase-polymerase chain reaction (qRT-PCR) assay described here targets the intergenic region between the F and G proteins (Fig 1B), which are only present in full-length virus genomes. This assay is a useful tool for quantifying NiV replication *in vitro* and *in vivo* and provides investigators with the ability to accurately monitor viral genomic titers in real time while studying potential sites of viral replication and development of medical countermeasures.

## Materials and methods

### Viral titration

NiV-M (accession KY425646) and NiV-B (accession AY988601) virus stocks were cultured following conditions previously described [18]. Virus stocks were titrated on Vero E6 cells (BEI Resources, Manassas, VA catalog # NR 596), in a biosafety level-4 (BSL-4) facility at the NIAID Integrated Research Facility in Frederick, MD. Ten-fold serial dilutions were performed, using plaque assay media (α-Minimal Essential Medium (α-MEM) with GlutaMAX (GIBCO, Life Technologies Carlsbad, CA), 5% heat-inactivated fetal bovine serum (GIBCO), and 1X antibiotic-antimycotic (penicillin, streptomycin and amphotericin B, GIBCO). Using a 6-well plate, Vero E6 cell monolayer was incubated with 300 μL of diluted virus, in triplicate, at 37°C with 5% CO_2_ for 60 min with gentle rocking every 10 min, and subsequently overlaid with 1X Avicel plaque media (1.25% Avicel RC-591; FMC Biopolymer, Philadelphia, PA), 1X α-MEM, Temin’s modification (GIBCO), 5% heat-inactivated fetal bovine serum, 1X antibiotic-antimycotic, and 1X GlutaMAX-I (GIBCO), and incubated at 37°C with 5% CO_2_ for 3 days. Following incubation, the semisolid Avicel overlay was removed, and the cell monolayers were subsequently stained with crystal violet dye (final concentration of 0.2% Gentian violet [Ricca Chemical, Arlington, TX] in 10% neutral-buffered formalin). The crystal violet dye was removed, the plates were washed with water, and the plaques were enumerated.

### Nucleic acid extraction

NiV virus stocks were 10-fold serially diluted in Dulbecco’s Modified Eagle Medium (GIBCO), treated with Trizol LS (Thermo Fisher Scientific, Waltham, MA) following manufacturer’s instructions, and removed from the BSL-4 laboratory. Viral and cellular RNA were extracted from Trizol LS treated samples using the QIAamp Viral RNA mini kit (Qiagen, Germantown, MD) following the manufacturer’s protocol. Extracted RNA was stored at -80°C until use.

### Primer probe design, PCR amplification and detection

Primers were designed against the prototype NiV-M (GenBank accession number AF212302) and NiV-B (GenBank Accession number AY988601) sequences and targeted the intergenic region between the F and G proteins. Primers specific for NiV-M and NiV-B include a forward primer recognizing nucleotides in the UTR of the *F* gene and a reverse primer recognizing nucleotides in the 3’ region of the genomic strand of the *G* gene. NiV-M primer sequences span nucleotides 8660–8686 (5’CCGTGAATATGTAATTGATAATTTCCC3’) (Integrated DNA Technologies (IDT), Coralville, IA) and 8727 to 8758 (5’GCTTAGAAAGATACAGTTAAGTATCCAATGA3’) (IDT). NiV-B primer sequences span nucleotides 8658 to 8682 (5’ GTACTCAACCATGAATGAACAGTTG 3’) (IDT) and 8735 to 8763 (5’CTTTAAAGGACACAGTTTAATATCCAATG3’) (IDT). The hydrolysis probe, with a 6-carboxyfluorescein (FAM) reporter with a minor grove binder, targets both NiV-M and NiV-B, spanning nucleotides 8707 to 8727 or 8713 to 8732, respectively, (FAM-5’CTTAGGACCCAGGTCCATAA3’-minor groove binder) (Applied Biosystems Inc., Thermo Fisher Scientific) in the 3’ region of the genomic strand of the *G* gene. Primers to target the *F* and *G* gene of NiV-M and NiV-B are described in S1 Table.

To generate RNA transcripts to serve as material for the standard curve of cycle threshold versus RNA concentrations, NiV-M nucleotides 8560 to 8859 or NiV-B nucleotides 8551 to 8867 (S1 Table) were synthesized in a pCMV6-AC vector (Blue Heron Biotech, Bothell, WA) under a T7 promoter and terminator. T7 transcription (MEGAscript T7 Transcription Kit, Thermo Fisher Scientific) generated synthetic RNA transcripts of the anti-genome, and these transcripts were purified using a RNeasy Mini kit (Qiagen) and quantified using a NanoDrop 8000 (Thermo Fisher Scientific). The copy number (x) of the synthetic RNA was calculated using the following formula:

$$x=\frac{RNA\;(ng)\times 6.022\;x\;10^{23}[Avogadro^{\prime }s\;number]}{Length\;(bp)\times 349\;[average\;nucleotide\;molar\;mass]\times 1\times 10^{9}}$$ (1)

The RNA transcripts were diluted to a concentration of 1 x 10^10^ copies/mL, aliquoted, and stored at -80°C.

qRT-PCR was performed using QuantiFast Pathogen RT-PCR kit (Qiagen) following the manufacturer’s recommendations, in combination with the previously identified primers and probe. The primers were used at a concentration of 1.5 μM per 25 μL reaction, and the probe was used at a concentration of 250 nM per 25 μL reaction. For each 25 μL reaction, 5 μL of extracted viral RNA were used. A one-step reaction was performed with the following cycling conditions using ABI 7500, an ABI 7900 real-time PCR system (Thermo Fisher Scientific) and a LightCycler 96 system (Roche, Indianapolis, IN) in independent assays. The reverse transcription step was at 48°C for 20 min, followed by an inactivation step at 95°C for 5 min. The qPCR step was set at 95°C for 15 s, followed by incubation at 58°C for 30 s, for 40 cycles. The fluorescence data was set to collect during the 58°C-amplification step. The ABI 7500 software was used to analyze all qRT-PCR results, to calculate the R^2^ and to determine the coefficient of correlation of the assay. For analysis, the baseline was set to ‘Auto baseline,’ and the threshold was set in the exponential phase of the amplification curve.

### Limit of detection

Studies were performed to determine the lowest detectable concentration (limit of detection, LoD) of extracted NiV-M and NiV-B viral RNA. NiV was diluted 10-fold serially with dilutions starting from 1 x 10^5^ PFU/mL to 1 x 10^−2^ PFU/mL. After identifying an initial LoD, quarter-log dilutions were generated from 1 x 10^2^ PFU/mL to 1 PFU/mL to determine the lowest detectable target concentration. Diluted NiV was treated with Trizol LS (Thermo Fisher Scientific) following manufacturer’s instructions, removed from the BSL-4 laboratory, and viral RNA was extracted from three replicates using the QIAamp Viral RNA mini kit (Qiagen) following the manufacturer’s protocol. qRT-PCR was performed using the extracted RNA in triplicate per each replicate, and the LoD was determined to be the lowest concentration at which all triplicates from each replicate were detected. The LoD for genome copies was calculated using the identified PFU:genome ratios.

### Amplification of viral mRNA

cDNA synthesis was performed (SuperScript VILO Master Mix, Thermo Fisher Scientific) to amplify viral and cellular polyadenylated RNA following the manufacturer’s instructions. The cDNA was subsequently treated with RNase to degrade all viral RNA to prevent amplification during qPCR. The cDNA and the primer-probe combination outlined above and a primer-probe combination that targets the nucleocapsid were used to perform qPCR [6].

### Specificity and cross-reactivity

Viral and cellular RNA from a panel of human and non-human cell lines including Vero E6 cells (African Green Monkey; kidney, ATCC, CCL-81, Manassas, VA and BEI Resources, NR-596, Manassas, VA), MA104 cells (African Green Monkey; kidney, ATCC, CRL-2378.1), BS-C-1 (African Green Monkey; kidney, ATCC, CCL-26), 293T cells (Human; kidney, ATCC, CRL-11268), and CHO-K1 cells (Chinese hamster; ovary, ATCC, CCL-61) were used for examining cross-reactivity. The panel of viruses examining cross-reactivity included NiV-B assay with NiV-M RNA, and the NiV-M assay with NiV-B RNA, as well as against Hendra virus (KY425627), Measles virus, Respiratory syncytial virus (GenBank Accession number KT992094; [19]) Ebola virus (GenBank Accession number KX000398), and Lassa virus (Josiah strain) (GenBank Accession numbers KY425632 and KY425638). All samples were tested in triplicate.

### Electron microscopy

For negative staining analysis and viral quantitation, cell culture supernatant was collected from infected Vero E6 cells clarified by low speed centrifugation, and inactivated by diluting in an equal volume of 8.0% paraformaldehyde (PFA) (E.M. Sciences, Warrington, PA), in Millonig’s sodium phosphate buffer (Tousimis Research, Rockville, MD) for a final concentration of 4% PFA.

After inactivation, 50 μL of supernatant was mixed with an equal volume of latex beads (Structure Probe Inc., West Chester, PA) of a known concentration, and 2 μL of the supernatant/latex bead samples were mounted on individual 300 mesh copper grids reinforced with formvar resin and carbon, allowed to dry, and stained with 1.0% Phosphotungstic Acid (E.M. Sciences). Specimen grids were examined in a Tecnai Spirit Twin Transmission Electron Microscope, (FEI, Hillsboro, OR) operating at 80 kV.

For virus quantitation, 1000 latex beads were enumerated over eight different grid openings (average of 125 beads per opening), along with intact NiV particles. Using ratio formulas, the concentration of each viral supernatant per mL was then calculated.

### Statistics

Coefficient of variation between experimental replicates and when identifying genome:PFU ratios for NiV-M and NiV-B was calculated using the following formula:

$$CV=\frac{Standard\;Deviation}{Mean}$$

## Results

### Analytical performance

Upon paramyxovirus infection, viral transcription occurs, producing a large amount of viral mRNA. These viral mRNA transcripts are present in varying amounts; N transcripts are the most abundant, and L transcripts are the least abundant (Fig 1A). To better understand NiV replication kinetics, we designed a one-step qRT-PCR assay that will target only the viral genomic RNA and not amplify viral mRNA transcripts. We designed the sense primer to anneal to the 5’ UTR of the *F* gene and the antisense primer to anneal within the 3’ coding region of the *G* gene. The hydrolysis probe also is designed to anneal to the 3’ UTR of the *G* gene. While this design does not prevent the antisense primer from using viral mRNA transcripts as templates for cDNA synthesis, the design does prevent the synthesized cDNA from serving as a template for qPCR (Fig 1B).

**Fig 2:**
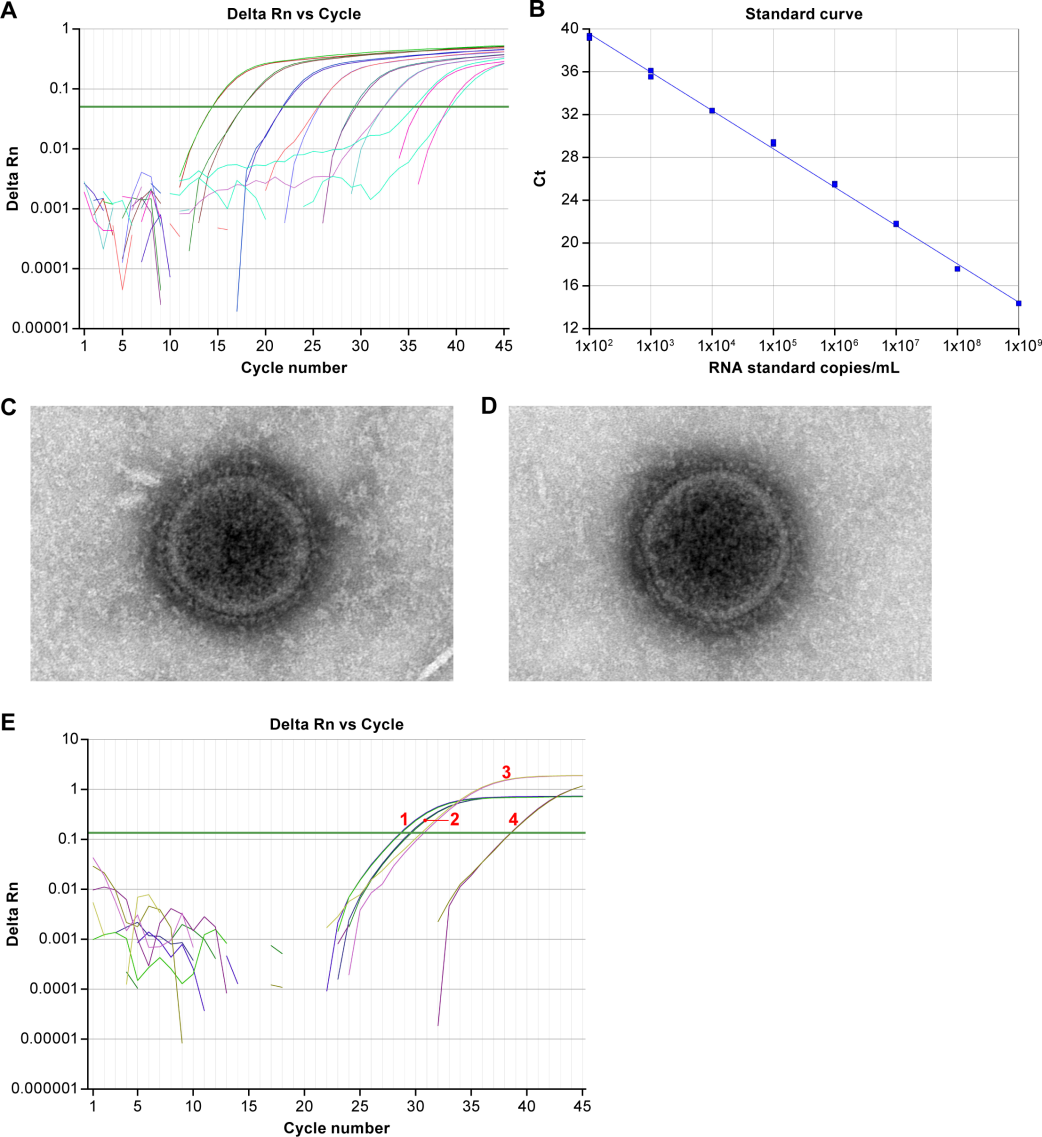
Amplification of T7 generated RNA synthetic transcripts. (A) Amplification plot of synthetic RNA transcripts that serve as the RNA standard shows the variation of the log of ΔRn (change in normalized reporter from baseline) with the cycle number. The green line is the threshold line, and the cycle threshold (Ct) is the intersection of the amplification curve and the threshold line. The copy number of the RNA standard in 1-log10 dilutions ranges from 1 x 10^2^ to 1 x 10^9^ copies/ml. (B) The standard curve has a slope of -3.58 and a r^2^ of 0.998. (C) Electron micrograph of Nipah virus Malaysia. (D) Electron micrograph of Nipah virus Bangladesh. (E) Detection of NiV-M mRNA transcripts. cDNA was generated from viral genomic RNA and mRNA transcripts using either a random nanomer or oligodT primer. qPCR was subsequently performed using primers/probe designed against the F-G intergenic region or a region within the N gene. Results demonstrate that the primers and probe designed against the N gene detected both genomic RNA (1) (average Ct value = 28.6) and mRNA transcripts (2) (average Ct value = 29.5), with mRNA transcripts significantly contributing to detectable total viral RNA levels. The primers and probe designed against the intergenic region detect the random nanomer cDNA at lower concentrations (3) (average Ct value = 30.6). Viral mRNA transcripts contribute an insignificant amount to measurable NiV-M RNA concentrations (4) (average Ct value = 38.5).

Assay efficiency was examined by using T7-derived synthetic RNA transcripts to generate a standard curve of cycle threshold versus RNA concentrations (Fig 2A and 2B). The RNA standard was assessed by serially diluting the standard 10-fold and performing the qRT-PCR assay. The amplification of the transcripts had a linear detection range from 1 x 10^2^ to 1 x 10^9^ copies/mL and had a coefficient of correlation of r^2^ = 0.998.

We also compared the NiV-M and NiV-B primer and probe sets designed against the intergenic region to primer and probe sets designed to target the coding region of the *F* and *G* gene. NiV-M and NiV-B was serially passaged to generate daughter stocks that were subsequently used for qRT-PCR analysis. For both NiV-M and NiV-B, the F and G primer probe sets were more sensitive due to the amplification of viral mRNA transcripts (Table 1).

**Table 1:** NiV assay comparison.

Virus	Virus Stock	Assay	Ct Values	Mean Viral RNA copies/mL	Coefficient of Variation
NiV-M	Daughter A	NiV-M Assay	22.8, 24.1, 22.6	7.87 x 10^10^	0.35
		NiV-M-F Assay	14.8, 15.2, 15.0	3.87 x 10^12^	0.15
		NiV-M-G Assay	15.1, 15.0, 15.2	3.72 x 10^12^	0.26
	Daughter B	NiV-M Assay	23.0, 23.0, 22.5	8.99 x 10^10^	0.10
		NiV-M-F Assay	15.3, 15.2, 15.3	3.43 x 10^12^	0.04
		NiV-M-G Assay	16.0, 15.7, 15.9	2.57 x 10^12^	0.03
	Daughter C	NiV-M Assay	20.8, 21.9, 20.81	2.03 x10^11^	0.05
		NiV-M-F Assay	12.9, 12.7, 12.8	1.13 x 10^13^	0.09
		NiV-M-G Assay	13.5, 13.5, 13.3	8.32 x 10^12^	0.04
NiV-B	Daughter A	NiV-B Assay	21.5, 21.4, 21.5	3.06 x 10^10^	0.03
		NiV-B-F Assay	15.4, 15.5, 15.5	1.01 x 10^12^	0.05
		NiV-B-G Assay	15.5, 15.6, 15.5	9.67 x 10^11^	0.05
	Daughter B	NiV-B Assay	21.9, 21.8, 21.9	2.42 x 10^10^	0.04
		NiV-B-F Assay	15.8, 15.9, 15.9	8.02 x 10^11^	0.05
		NiV-B-G Assay	16.0, 15.9, 15.9	7.85 x 10^11^	0.03
	Daughter C	NiV-B Assay	22.6, 22.7, 22.5	1.59 x 10^10^	0.07
		NiV-B-F Assay	16.5, 16.6, 16.5	5.44 x 10^11^	0.03
		NiV-B-G Assay	16.5, 16.6, 16.6	5.45 x 10^11^	0.03

NiV-M and NiV-B was passaged 3 consecutive times and a qRT-PCR was performed on each sample in triplicate using the assay that targets the F/G intergenic region, the F coding region or the G coding region. The coefficient of variation of the mean viral RNA copies/mL was calculated for each daughter sample analyzed.

### Genome to PFU to particle correlation

The ratio of viral genomes to PFU during NiV infection was determined for both NiV-M and NiV-B. NiV-M and NiV-B stocks (1.72 x 10^6^ PFU/mL and 3.38 x 10^6^ PFU/mL, respectively) were titrated to 1 x 10^6^ PFU/mL, and 1-log_10_ dilutions were generated from 1 x 10^5^ PFU/mL to 1 x 10^−2^ PFU/mL. Each dilution point was treated as an individual sample, and the titer was determined by plaque assay to confirm the PFU concentration of each sample. The concentration of viral genomes/mL was determined by qRT-PCR analysis for each sample, and the ratio of viral genomes/mL to PFU/mL was determined. The results indicate that NiV-M and NiV-B isolates have a genome to PFU ratio of approximately 1000:1 (Tables 2 and 3). In addition, the NiV-M and NiV-B parent stocks used to calculate the genome to PFU ratio were passaged 5 consecutive times to generate daughter stocks. The titer for each daughter sample was determined by plaque assay and qRT-PCR was performed to calculate the concentration of viral genomes/mL. The results were consistent with our previous results indicating that NiV-M and NiV-B have a genome to PFU ratio of approximately 1000:1 (Table 4).

**Table 2:** Calculation of NiV-M genome to PFU ratio.

Target titer (PFU/mL)	Unadjusted qRT-PCR (genomes/mL)	qRT-PCR dilution factor	Genomes/mL	PFU/mL*	Genome:PFU Ratio	Mean Genome:PFU Ratio	Coefficient of Variation
1.00 x 10^6^	1.94 x 10^6^	800	1.55 x 10^9^	1.50 x 10^6^	1.04 x 10^3^	1.04 x 10^3^	0.189
1.00 x 10^5^	2.03 x 10^5^	800	1.62 x 10^8^	1.24 x 10^5^	1.31 x 10^3^		
1.00 x 10^4^	1.72 x 10^4^	800	1.37 x 10^7^	1.40 x 10^4^	9.81 x 10^2^		
1.00 x 10^3^	1.43 x 10^3^	800	1.14 x 10^6^	1.36 x 10^3^	8.40 x 10^2^		

PFU, plaque forming unit; qRT-PCR, quantitative reverse transcriptase polymerase chain reaction

All assays were performed in triplicate for each sample. The coefficient of variation of the mean genome:PFU ratio was calculated from using the genome:PFU ratios from the individual target titer samples.

*Based on back titration of assayed stock

**Table 3:** Calculation of NiV-B genome to PFU ratio.

Target Titer (PFU/mL)	Unadjusted qRT-PCR (genomes/mL)	qRT-PCR Dilution Factor	Genomes/mL	PFU/mL*	Genome:PFU Ratio	Mean Genome:PFU Ratio	Coefficient of Variation
1.00 x 10^6^	2.24 x 10^6^	800	1.80 x 10^9^	1.23 x 10^6^	1.46 x 10^3^	1.63 x 10^3^	0.256
1.00 x 10^5^	1.99 x 10^5^	800	1.60 x 10^8^	1.28 x 10^5^	1.25 x 10^3^		
1.00 x 10^4^	2.28 x 10^4^	800	1.82 x 10^7^	1.16 x 104	1.58 x 10^3^		
1.00 x 10^3^	3.28 x 10^3^	800	2.62 x 10^6^	1.18 x 10^3^	2.22 x 10^3^		

PFU, plaque forming unit; qRT-PCR, quantitative reverse transcriptase polymerase chain reaction

All assays were performed in triplicate for each sample. The coefficient of variation of the mean genome:PFU ratio was calculated from using the genome:PFU ratios from the individual target titer samples.

*Based on back titration of assayed stock

**Table 4:** Passaged NiV genome to PFU ratio.

Virus	Virus Stock	Calculated Titer	Calculated Viral RNA copies/mL	Genome:PFU ratio	Mean Genome:PFU ratio	Coefficient of Variation
NiV-M	Passage 1	3.89 x 10^7^	7.87 x 10^10^	2.02 x 10^3^: 1	1.40 x 10^3^: 1	0.417
	Passage 2	4.33 x 10^7^	8.61 x 10^10^	1.99 x 10^3^: 1		
	Passage 3	9.22 x 10^7^	1.17 x 10^11^	1.27 x 10^3^: 1		
	Passage 4	1.27 x 10^8^	8.99 x 10^10^	7.08 x 10^2^: 1		
	Passage 5	1.97 x 10^8^	2.03 x 10^11^	1.03 x 10^3^: 1		
NiV-B	Passage 1	1.97 x 10^7^	3.06 x 10^10^	1.55 x 10^3^: 1	1.53 x 10^3^: 1	0.212
	Passage 2	1.28 x 10^7^	2.42 x 10^10^	1.89 x 10^3^: 1		
	Passage 3	1.42 x 10^7^	1.59 x 10^10^	1.12 x 10^3^: 1		
	Passage 4	1.78 x 10^7^	3.20 x 10^10^	1.80 x 10^3^: 1		
	Passage 5	2.36 x 10^7^	3.06 x 10^10^	1.30 x 10^3^: 1		

NiV-M and NiV-B were passaged 5 consecutive times and a titer and genome concentration was calculated from each sample. A mean genome:PFU ratio and the coefficient of variation was calculated from the 5 individual samples. All assays were performed in triplicate.

To determine the ratio of viral genomes and PFU to virus particles, particle counts from NiV-M and NiV-B stocks, calculated using electron microscopy, were 9.80 x 10^6^ and 1.04 x 10^7^ particles/mL respectively. Using the previously determined genome to PFU ratios and normalizing the ratio so that the PFU equals 1, we calculated the PFU:genome:particle ratio as 1:1.04 x 10^3^:5.69 for NiV-M and 1:1.63 x 10^3^:3.07 for NiV-B (Table 5).

**Table 5:** NiV-M and NiV-B genome:Particle:PFU ratio.

Virus	Genome	Particle	PFU
NiV-M	1.04 x 10^3^	5.69	1
NiV-B	1.63 x 10^3^	3.07	1

Electron microscopic evaluation found that NiV-M and NiV-B particles were consistent with the morphology of NiV and exhibited generally spherical overall forms, although some oval and pleomorphic-shaped particles were also observed. The virus particles exhibited internal capsomere structures and fringe envelope proteins on the viral envelope (Fig 2C and 2D).

We also demonstrated that viral mRNA transcripts were not amplified during the cDNA synthesis step, thereby contributing to the overall viral RNA concentrations. An oligodeoxythymidine (oligodT) primer was used to amplify NiV-M mRNA transcripts that were polyadenylated, and a random nanomer primer was used to amplify both NiV-M genomic RNA and mRNA transcripts at a concentration of 1 x 10^6^ PFU/mL. Amplified cDNA was subsequently used for qPCR using either the primer-probe set designed against intergenic region or the primer-probe set that was specific for the *N* gene [6]. Results demonstrate that the N-specific primer-probe set detected NiV-M viral RNA at an earlier cycle threshold (increased sensitivity) than the NiV-M intergenic primer-probe set when cDNA was generated from either a random nanomer primer or an oligodT primer (Fig 2E). While the intergenic primer-probe set did detect cDNA generated from using oligodT primer (Fig 2E, curve 3), the cycle threshold at which the RNA was detected indicates that viral mRNA would contribute an insignificant amount to the overall concentrations of viral genomic RNA (Fig 2E, curve 4). One possible explanation for the observed detection of viral mRNA is that low levels of bi- or polycistronic viral mRNA were generated from read-through transcription, and detected by our primer-probe set. In addition, we cannot rule out that the oligodT primer was priming from the viral genomic RNA at very low concentrations.

### Limit of detection

The limit of detection (LoD) of the NiV-M and NiV-B assays was determined by identifying the lowest concentration of NiV at which genomic material in all replicates was detected. Log dilutions of viral stocks were generated ranging from 1 x 10^5^ PFU/mL to 1 x 10^−2^ PFU/mL, with approximate genome copies/mL calculated based off the identified PFU:genome ratio. Viral RNA was extracted from three replicate samples for each dilution and examined by qRT-PCR in triplicate. After identifying an initial LoD, quarter-log dilutions were generated from 1 x 10^2^ PFU/mL to 1 PFU/mL. The LoD of the NiV-M qRT-PCR assay was determined to be 5.6 PFU/mL or 5.82 x 10^3^ genomes/mL. The LoD of the NiV-B assay was 10 PFU/mL or 1.63 x 10^4^ genomes/mL (Table 6).

**Table 6:** Determination of the limit of detection of the NiV-M and NiV-B qRT-PCR.

Assay	Target Titer (PFU/mL)	Calculated Viral RNA copies/mL	Replicate Ct Values/RNA Extraction		
			1	2	3
NiV-M	10	1.04 x 10^4^	37.5, 37.4, 37.2	37.2, 36.4, 37.4	36.9, 37.1, 37.2
	5.6	5.82 x 10^3^	39.6, 39.2, 39.	39.8, 38.4, 38.3	39.2, 38.5, 38.5
	3.1	3.22 x 10^3^	38.8, 39.2, 38.8	39.0, 39.9, 38.4	38.0, ND, 39.6
	1.7	1.77 x 10^3^	ND, 39.6, ND	39.6, ND, ND	ND, ND, ND
	1.00	1.04 x 10^3^	ND, ND, 36.9	ND, ND, ND	ND, ND, ND
NiV-B	10	1.63 x 10^4^	38.6, 36.6, 37.5	36.9, 37.0, 36.8	36.7, 36.7, 38.1
	5.6	9.13 x 10^3^	38.3, 38.5, 38.2	38.9, 39.9, ND	ND, 39.1, 39.0
	3.1	5.05 x 10^3^	ND, 39.4, ND	39.1, 39.1, 39.1	39.3, 38.0, 39.6
	1.7	2.77 x 10^3^	ND, ND, ND	ND, ND, ND	ND, ND, ND
	1.00	1.63 x 10^3^	ND, ND, ND	ND, ND, ND	ND, ND, ND

Ct, cycle threshold; ND, not detected

### Cross reactivity

Assay specificity was examined to confirm that neither assay cross reacts to other NiV variants, Hendra virus (HeV), Measles virus (MeV), Respiratory Syncytial Virus (RSV), Ebola virus (EBOV), or Lassa virus (Josiah strain) (LASV), and host cell mRNA (Table 7). Viral RNA was isolated from stock viruses. Cellular RNA was extracted from several different cell lines representing possible sample sources, including human (293T), Chinese hamster (CHO), and African green monkeys (BSC-1, MA104, Vero E6) cells and used as a template for qRT-PCR. The results indicate that the NiV intergenic qRT-PCR assay does not amplify host genomic material from any of the cell types examined (Table 7).

**Table 7:** Determination of cross-reactivity of the NiV-M and NiV-B assays.

	Test Sample	NiV-M Assay	NiV-B Assay
**Virus Stocks**	NiV-M	D	ND
	NiV-B	ND	D
	HeV	ND	ND
	MeV	ND	ND
	RSV	ND	ND
	EBOV	ND	ND
	LASV	ND	ND
**Cell Lines**	293T	ND	NT
	Vero E6	ND	ND
	MA104	ND	NT
	CHO	ND	NT
	NR596	ND	ND
	BSC1	ND	NT

D, Detected; ND, not detected; NT, not tested

## Discussion

We developed a novel one-step NiV qRT-PCR assay that measures viral genomic RNA with minimal contribution from NiV mRNA transcripts (Fig 1). Additionally, the probe was designed against a conserved sequence in the *G* gene for use with primer sets specific to known NiV isolates, decreasing the need to design multiple probes when working with NiV variants. As new NiV variants are identified, the ability of the probe to recognize a conserved sequence could provide time and cost benefits over designing and developing novel assays for each variant. However, the intent of this assay is to provide effective quantification of virus genome, not as a tool for diagnostic or epidemiological studies. By limiting the contribution of viral mRNA transcripts, we demonstrated that NiV-M and NiV-B have a PFU to genome to particle ratios of approximately 1:1.04 x 10^3^:5.69 and 1:1.63 x 10^3^:3.07, respectively.

Due to the assay targeting only viral genomic RNA, we expected our assay to have reduced sensitivity and higher limit of detection when compared to traditional one-step qRT-PCR assays previously used. Indeed, we demonstrated that when our assay was compared to a published assay [20], we did see reduced sensitivity (Fig 2E). As the N-specific PCR assay detects all available viral RNA, the reduced sensitivity in the genome specific assay is due to less viral RNA/RNA transcripts for our assay to target. Therefore, the genome-specific NiV PCR assay would not be an ideal diagnostic tool due to reduced sensitivity.

The probe used for both the NiV-M and NiV-B assays was designed against a conserved sequence in 3’ region of the *G* gene. Since the same probe can be used interchangeably between the NiV-M or NiV-B assays, demonstration of no cross-reactivity between each assay and non-targeted NiV variants is important. Both assays were shown to only detect the targeted viral variant. The NiV-M assay does not cross react with NiV-B viral RNA, and the NiV-B assay does not cross react NiV-M viral RNA. Additionally, neither assay cross reacts with the closely related henipavirus, Hendra virus, or other tested (-)sense RNA viruses, including Ebola virus, Lassa virus (Josiah strain), or other paramyxoviruses including measles virus and respiratory syncytial virus. The assay also does not cross-react with potential contaminating material including extracted RNA from uninfected human, non-human primate, and rodent cell lines.

By targeting the Nipah virus intergenic region and separating the contribution of viral mRNA transcripts from viral genomic RNA we can more accurately quantify virus concentration than with traditional one-step qRT-PCR assays. This assay can provide a more precise measurement of viral load reduction than traditional one-step qRT-PCR assays when screening vaccine candidates and potential therapeutics.

## Supporting information

S1 Table Standard RNA target sequences, with the corresponding genome position, and sequences for the primers and probes used in the NiV specific assays. (DOCX)
