# Supplementary material for: Development of a novel real-time polymerase chain reaction assay for the quantitative detection of Nipah virus replicative viral RNA
Source: PLoS One. 2018 Jun 19;13(6):e0199534. doi: 10.1371/journal.pone.0199534 (PMC6007899; doi:10.1371/journal.pone.0199534)
Supplement: S1 Table — (DOCX) [file pone.0199534.s001.docx]

**S1 Table. NiV Standard Sequences and Primer Probe sequences**

| **NiV RNA Standard Sequences** | | | | |
| --- | --- | --- | --- | --- |
| **Virus** | **Genome Position** | | **Sequence** | |
| NiV-M | 8560-8859 | | 5’-ATAATGACAAATAAGGATTCAAATTGGATTATGATATA GTTTCATACTACAATAGCATTTCGACCAAGAAAATATCCTTACAATTATACAATGTACTTAACCGTGAATATGTAATTGATAATTTCCCTTTAGAAATTTAATAAAAAACTTAGGACCCAGGTCCATAACTCATTGGATACTTAACTGTATCTTTCTAAGCTATCACATATCAAAGGAGAGATTGAATGCTTTTTTGGAGATCTAGATCATTACTATATGTGTCTCCTATAATCACATCATAGGAGTGAACCATAATACACA-3’ | |
| NiV-B | 8551-8867 | | 5’-CTCATAATTAATGATATGATGATAAATAAAGATTCAA ATTGACTTATGATATAATTTCATACTATAACAACATCTCAACCAAGGAAATGTACTTGCAATTATACATTGTACTCAACCATGAATGAACAGTTGATATTCTTTTTTTGAAAAAATTATAAAAAACTTAGGACCCAGGTCCATAACTCATTGGATATTAAACTGTGTCCTTTAAAGTTATCATATATCAGGGGAGAAATAAAAGGTTTTTTAAGGTTCTTAGATCATCATAATATGTATCTTCTTTAATCACATTATAGGAGTGAGCCACAATATACATC-3’ | |
| **Primer and Probe Sequences** | | | | |
| **Name** | | **Target Virus** | **Nucleotide Position** | **Sequence** |
| NiV-Mal F-FWD | | NiV-M | 7653-7673 | 5’ACAAAGAGGAGCGTGATCTGC3’ |
| NiV-Mal F-REV | | NiV-M | 7716-7739 | 5’CTTCTCAGTCGATCCCGTTAAACA3’ |
| NiV-Mal F-Probe | | NiV-M | 7685-7710 | FAM-5’TGCCACACCTATGACCAACAACATGA3’-MGB |
| NiV-Mal G-FWD | | NiV-M | 9895-9916 | 5’GTCTAGCTGTGAAACCCAAGAG3’ |
| NiV-Mal G-REV | | NiV-M | 9984-10005 | 5’CTGAAGGTCCATACGGCATAAC3’ |
| NiV-Mal G-Probe | | NiV-M | 9947-9972 | FAM-5’TGCCCTACGAAGTATCGAGAAAGGGA3’-MGB |
| NiV-Bang F-FWD | | NiV-B | 7760-7781 | 5’GGTTGTTTCATCACACGTTCCC3’ |
| NiV-Bang F-REV | | NiV-B | 7842-7864 | 5’GAGATTGCCCTACCTGTTGTTTG3’ |
| NiV- Bang F-Probe | | NiV-B | 7817-7840 | FAM-5’TTGCATAAGCGTCACATGCCAGTG3’-MGB |
| NiV- Bang G-FWD | | NiV-B | 9743-9765 | 5’GTTTATGACTAACGTCTGGACCC3’ |
| NiV- Bang G-REV | | NiV-B | 9818-9841 | 5’GACACTGCACAAAGCACATAATAG3’ |
| NiV- Bang G-Probe | | NiV-B | 9777-9800 | FAM-5’AACACCGTTTACCATTGCAGTGCC3’-MGB |
